# Prognostic Value and Predication Model of Microvascular Invasion in Patients with Intrahepatic Cholangiocarcinoma

**DOI:** 10.7150/jca.32199

**Published:** 2019-09-07

**Authors:** Zheng Tang, Wei-Ren Liu, Pei-Yun Zhou, Zhen-Bin Ding, Xi-Fei Jiang, Han Wang, Meng-Xin Tian, Chen-Yang Tao, Yuan Fang, Wei-Feng Qu, Zhi Dai, Shuang-Jian Qiu, Jian Zhou, Jia Fan, Ying-Hong Shi

**Affiliations:** 1 Department of Liver Surgery, Liver Cancer Institute, Zhongshan Hospital, Fudan University; Key Laboratory of Carcinogenesis and Cancer Invasion of Ministry of Education, Shanghai, China; 2 Institutes of Biomedical Sciences, Fudan University, Shanghai, People's Republic of China

**Keywords:** Microvascular invasion, Intrahepatic Cholangiocarcinoma, Propensity score matching, Nomogram, Survival Analysis

## Abstract

**Background**: Whether microvascular invasion (MVI) adversely influences oncological outcomes for intrahepatic cholangiocarcinoma (ICC) patients remains unclear. The purpose of this study was to determine the impact of MVI on postoperative survival and establish a new predictive model for MVI before surgical intervention in patients with ICC.

**Methods**: In this two-center retrospective study, 556 and 31 consecutive patients who underwent curative liver resection for ICC at ZSH and XJFH were analyzed, respectively. Propensity score matching (PSM) and Cox regression analyses were used to explore the prognostic role of MVI on the OS and DFS. Multivariate logistic regression was used to identify the relative risk factors of MVI, which were incorporated into the nomogram.

**Results**: After PSM, 50 MVI cases matched with 172 non-MVI cases, and no bias was observed between the two groups (propensity score, 0.118 (0.099, 0.203) *vs.* 0.115 (0.059, 0.174), *p*=0.251). The multivariate Cox analysis showed that MVI was negatively associated with OS (HR 1.635, 95% *CI* 1.405-1.993,* p*=0.04) and DFS (HR 1.596, 95% *CI* 1.077-2.366,* p*=0.02). The independent factors associated with MVI were ALT, AFP, tumor maximal diameter, and tumor capsule. The nomogram that incorporated these variables achieved good concordance indexes for predicting MVI. Patients with a cutoff score of 168 were considered to have different risks of the presence of MVI preoperatively.

**Conclusions**: The presence of MVI was an adverse prognostic factor for ICC patients. Using the nomogram model, the risk of an individual patient harboring MVI was determined, which led to a rational therapeutic choice.

## Background

Intrahepatic cholangiocarcinoma (ICC) is a relatively rare cancer, accounting for 8-10% of cholangiocarcinomas (CCAs) and 5-30% of all primary liver malignancies, which arises from the endothelial cells of segmental or proximal branches of the bile duct 1. The newest statistic database showed the undoubted facts that ICC has risen steadily across the world over the past few decades with concomitant falls in extrahepatic cholangiocarcinoma (ECC) rates 2. However, the clinical outcome and treatment options have not improved remarkably with regard to the incidence increment 3. Therefore, an effective prediction for recurrence and the identification of key indicators for overall survival could tailor the initial therapeutic options, aiming to achieve the maximum benefit in ICC patients.

Microvascular invasion (MVI) is associated with adverse events in hepatocellular carcinoma (HCC), lung cancer and renal carcinoma in previous studies 4. In HCC, there is a correlation between a higher MVI incidence and a shorter disease-specific survival and recurrence-free survival 5. The presence of MVI may assist physicians in choosing the appropriate treatment, particularly for surgical resection margin consideration or adjuvant therapy for resectable patients 6,7, currently, few studies in ICC refer to MVI in terms of its prognostic value. ICC was reported to have locally aggressive behaviors, such as infiltration of the contiguous liver parenchyma, hepatic hilar and lymph node involvement 8, and microscopically invasive spread, with neural, perineural and lymphatic involvement 9. However, MVI is difficult to detect before pathological evaluation in ICC, even if recent superior imaging procedures were used during the patient evaluation. Therefore, there is a particularly important and urgent need to evaluate the clinical significance of MVI in ICC patients, establishing the predictive model of MVI in ICC patients, and identifying the recurrence risk after surgery as well.

The objective of the present study was to summarize multi-institutional clinical data and implement a propensity score matching (PSM) to investigate the association between MVI and long-term outcomes in ICC patients. Moreover, we developed a nomogram model based on preoperative clinical variables to predict the occurrence of MVI, which may be a useful tool for clinicians to choose optimal treatments for ICC patients.

## Methods

### Study Population and Criteria

Between January 31, 2000, and July 14, 2012, data on 701 consecutive patients who underwent curative surgery for pathologic histology confirmed ICC were prospectively collected at the Liver Cancer Institute, Zhongshan Hospital, Fudan University (ZSH). In addition, we obtained 31 ICC patients enrolled between January 2010 and May 2017 from the Fifth Affiliated Hospital of XinJiang Medical University (XJFH) as an external validation cohort. 556 eligible patients from ZSH were randomly assigned to training cohort (372 ICC patients) and validation cohort 1 (184 ICC patients, internal validation cohort) in 2:1 ratio by using the software R 3.3.2 with the random capture system, and 31 eligible patients from XJFH as validation cohort 2 (external validation cohort). Ethical approval was obtained from the Institutional Ethics Committee of the Zhongshan Hospital.

The exclusion criteria included to following: (*i*) 46 patients were excluded for preoperative transarterial chemoembolization (TACE), radiofrequency ablation (RFA), or radiotherapy and (*ii*) 97 patients were excluded for non-curative resection, recurrent lesions, and widespread metastasis, and (*iii*) 2 patients lacked pathological information or complete clinical. Overall, 556 and 31 patients were included in this study. The following clinical data and pathological results were collected: (1) demographic data, including age, gender, operative year; (2) results of preoperative laboratory blood tests, including HBsAg, Anti-HCV, AFP, ALT, AST, PT, CEA, and CA19-9; (3) imaging and pathological findings, including maximal diameter, tumor number, tumor capsule, tumor differentiation, MVI, lymph node invasion, ascites, and the presence of cirrhosis.

### Diagnostic criteria of microvascular invasion

The diagnostic criterion of MVI was the presence of a tumor cell nest in the vascular covered with endothelial cell, and only when the number of suspended tumor cell in the microvascular in excess of 50, it would be recorded as MVI under microscopic examination 10. Every specimen was reviewed independently by three pathologists to confirm the MVI diagnosis in the Zhongshan Hospital, Fudan University. If the three pathologists had an inconsistent diagnosis, the findings were discussed to reach a final decision.

### Data Source

The survival data was provided by the Liver Cancer Institute, Zhongshan Hospital, Fudan University, relying on the hospital medical records followed-up regularly at outpatient clinics or contacts with patients by phone. The OS was defined as the time from the surgery to death from any cause, and the DFS was defined as the time from the surgery to the first recurrence or death.

Before applying the PSM, we estimated the ideal sample size for comparing differences between the cohort groups. We found that the OS rates in the MVI and non-MVI groups were 3.3% and 35.4%, respectively. Assuming a type I error rate of 1% (α=0.01) and a power of 90% (β=0.1), 41 ICC patients per cohort group were needed to PSM and develop a predictive model. Assuming the incidence rates of MVI to be 25% in surgical specimens obtained after liver resection and transplantation 7, if the relative risk was 5, a 2-sided 5% significance level, 27 patients were required to achieve 90% power based on a test for external validation group.

### Statistics analysis

Statistical evaluation was conducted with SPSS 22.0 (SPSS, Chicago, IL) and R 3.3.2 software (www.r-project.org). The categorical variables were shown as whole numbers and proportions, and the continuous variables are described as the median with interquartile range as appropriate. Two-sided *p* < 0.05 were considered statistically significant. All confidence intervals (CIs) were stated at 95% confidence level.

PSM was used to reduce confounding 11-12. Logistic regression and multivariate Cox regression were used to find the confounders, which were based on Akaike's information criterion (AIC). The caliper was set at 0.05, and we used an optimal match ratio of 1:4. Mann-Whitney U test and Pearson Chi-Square Tests were used to analyze the difference between ICC patients before and after PSM.

The OS and DFS were calculated by the Kaplan-Meier method, and the difference of variables was compared using log-rank tests. Univariate cox regression and multivariate cox regression were used to examine the association between MVI and OS, DFS. The independent factors associated with MVI were formulated based on the results of the multivariate logistic regression analysis.

Nomogram for possible prognostic factors associated with MVI were established by R 3.3.2. The performance for predicting outcome was measured by the concordance index (C index) and calibration curves. Receiver operating characteristic curve (ROC) analysis that were determined by the Youden index, and the maximizing value of the Youden index was used to calculate the optimal cutoff values. Heat map was used to simplify the assessment of MVI risk.

## Results

### 556 ICC patients' baseline characteristics before PSM

**Figure 1:**
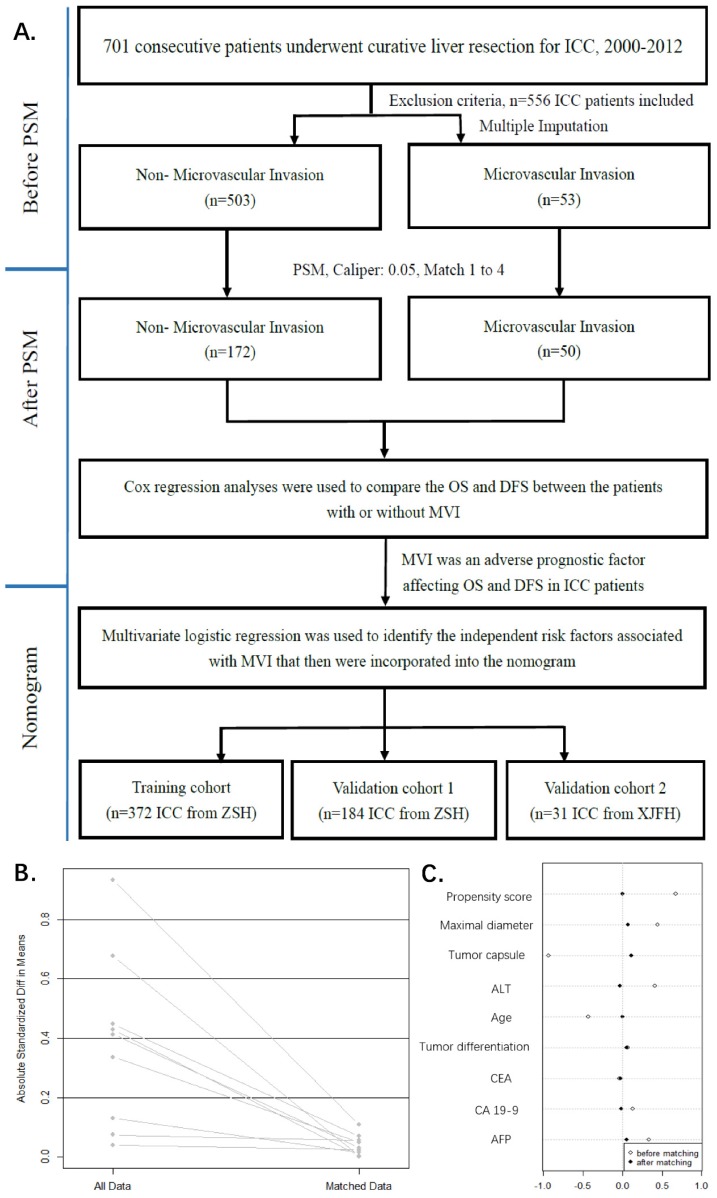
** Study flowchart.** ZSH, Zhongshan Hospital, Fudan University; XJFH, Fifth Affiliated Hospital of XinJiang Medical University (**A**). **The model values of standard mean differences (SMD) before and after PSM.** lineplot of standardized differences before and after PSM (**B**). Dotplot of SMD (Cohen's d) for all covariates before and after PSM (**C**). The SMD of propensity score and nine confounders (Propensity score, Maximal diameter, Tumor capsule, ALT, Age, Tumor differentiation, CEA, CA19-9, AFP) were matching. The SMD of matched data was depicted in rhombus dot.

The study flowchart is shown in **Figure 1A**. The study was censored on July 14, 2012. The median follow-up time of the 556 patients with ICC was 13 months (range, 1 to 134 months), and the end follow-up time was November 2015. Histopathologically identified 53 (9.53%) MVI-positive and 503 (90.47%) MVI-negative patients at our center. We described the patient demographics and clinical characteristics for the two groups (**Table 1**). The clinical data for 8 of the 19 variables differed significantly (*p*<0.05) as a result of a conspicuous bias, with a pre-described propensity score (PS, 0.066 (0.043, 0.115) *vs*. 0.124 (0.101, 0.203),* p*<0.001).

### 222 matched ICC patients' baseline characteristics after PSM

Confounders, major threat to the validity of this observational study were tumor capsule, maximal diameter, ALT, Age, tumor differentiation, CEA, CA19-9, AFP**,** which were selected by previous described procedures (**Figure 1C**). The propensity score matching procedure was performed to reduce the confounding variables based on the eight identified factors.

In PSM, we found 50 of the 53 MVI patients were matched with 172 of the 503 non-MVI patients. The propensity score suggests there were no biases in the matched groups (PS, 0.115(0.059, 0.174)) *vs*. 0.118(0.099, 0.203), *p*=0.251). In **Table 1**, the matched patient characteristics were compared, and no significant differences were shown between the groups, considering all 19 variables. **Figure 1B** shows a line plot of the standardized mean differences (SMD) and the SMD of all eight confounders, and the PS decreased to less than 0.2 after matching. **Figure 1C** shows a dot plot of the covariate balance in terms of the standardized mean differences for all the individual covariates, and the covariate balance improved in the matched data.

### Prognostic value of MVI

**Figure 2:**
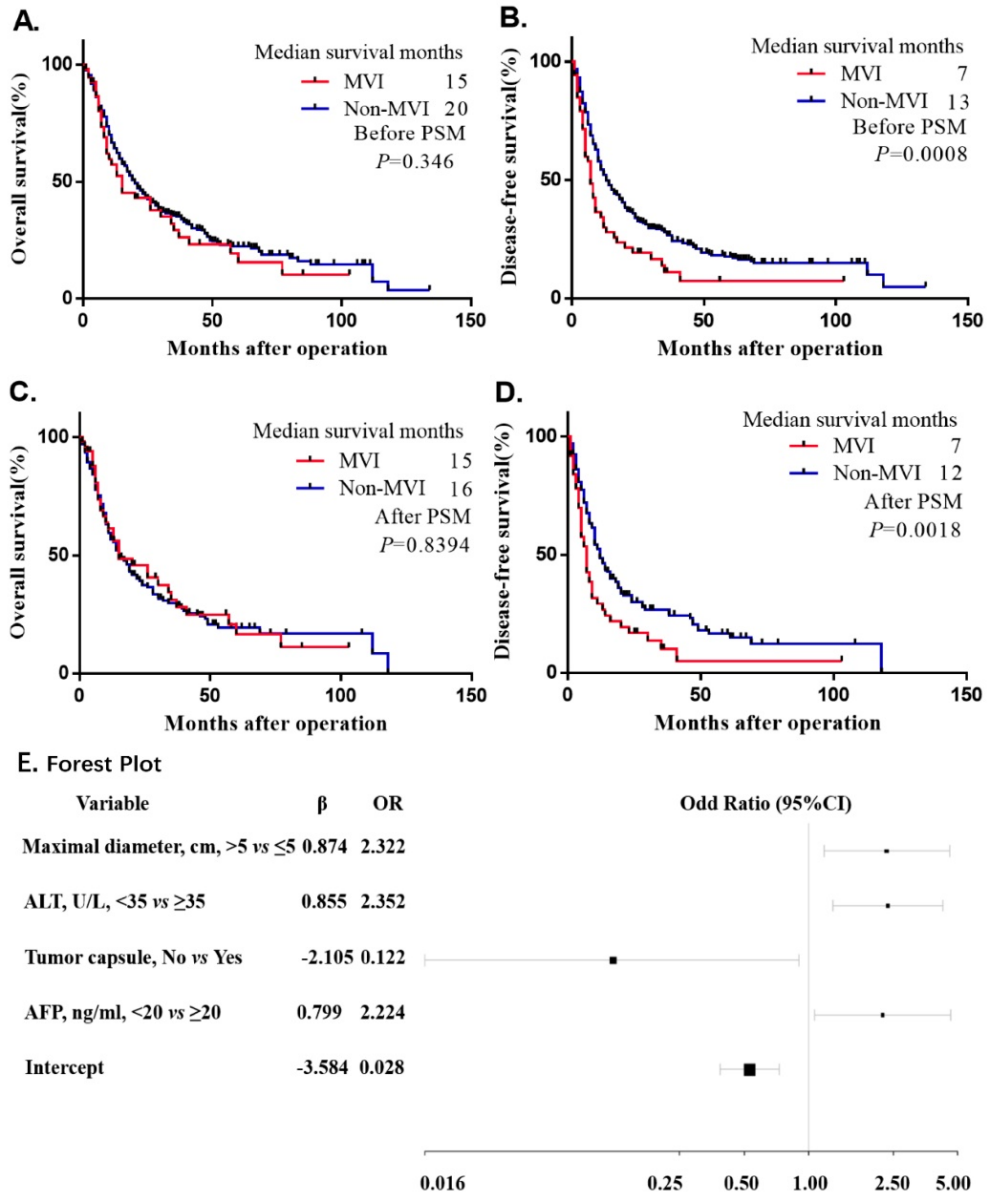
** Kaplan-Meier survival plot of OS and DFS before and after PSM.** The survival curve of overall survival and disease-free survival in unadjusted model (**A.B.**). The survival curve of overall survival and disease-free survival after matched (**C.D.**). **Multivariate Logistic Regression Analysis the risk factors of MVI Presence Based on Preoperative Data in the Training Cohort and forest plot drawn**. Abbreviations: ALT, alanine transaminase; AFP, alpha-fetoprotein; OR, odds ratio. Unstandardized β coefficients were calculated from the multivariate logistic regression model (**E**).

The univariate Cox proportional hazards regression analysis indicated that MVI had a negative influence on the DFS before and after PSM, which indicated a 67% risk of overall recurrence rate before matching (HR: 1.67, 95% *CI*: 1.223-2.281, *p*<0.001) and a 71.5% risk of overall recurrence rate after matching (HR: 1.715, 95% *CI*: 1.204-2.442, *p*=0.003). Nevertheless, no difference was found in the OS before and after PSM (*p*=0.354,* p*=0.842) (**Supplemental Table 1**). Additionally, the Kaplan-Meier curve of the DFS showed that the non-MVI group had a significant benefit compared with the MVI group (*p*=0.0008,* p*=0.0018) before and after PSM (**Figure 2B and Figure 2D**). Consistently, we did not observe significant difference in the Kaplan-Meier curve of the OS (*p*=0.346,* p*=0.8394) before and after PSM (**Figure 2A and Figure 2C**), Before PSM, MVI had a negative effect on the risk of DFS (HR: 1.414, 95% *CI*: 1.012-1.975, *p*=0.042). However, after PSM and performing a multivariate risk dependent Cox regression, we found that the OS (HR: 1.632, 95% *CI*: 1.405-1.993,* p*=0.04) or DFS (HR: 1.596, 95% *CI*: 1.077-2.366, *p*=0.02) was significantly different due to MVI (**Table 2**).

### Development and Validation of Nomogram for predicting MVI

**Figure 3:**
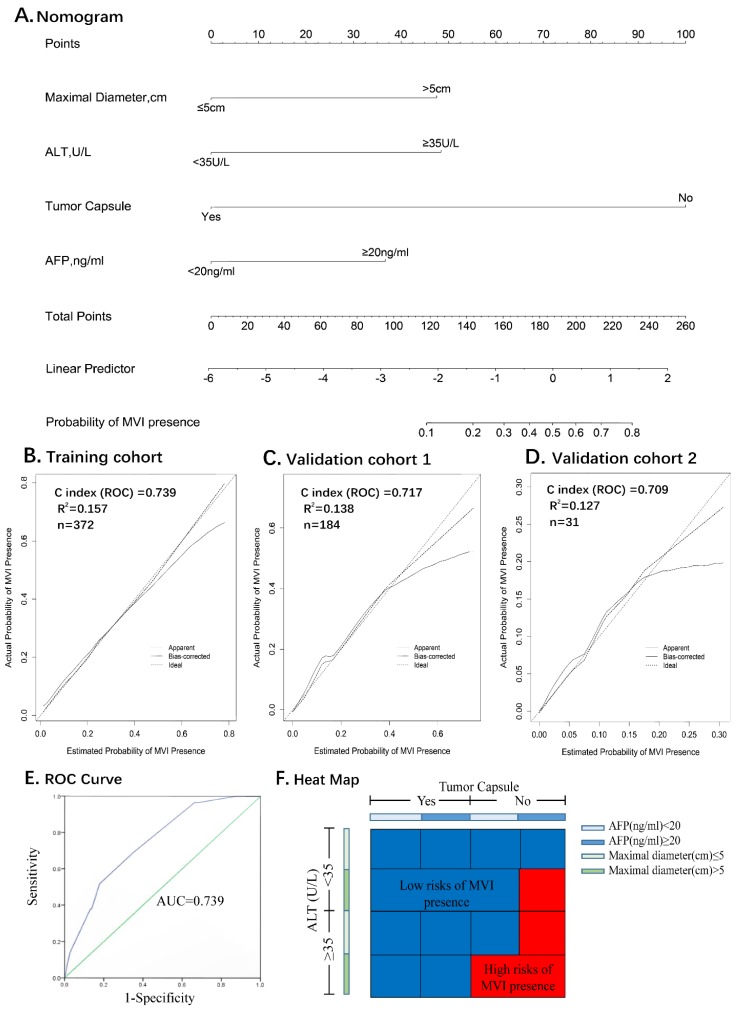
** Nomogram for Preoperative Estimation of Microvascular Invasion Risk and Its Predictive Performance.** Nomogram to estimate the risk of MVI presence preoperatively in ICC (**A**). Internal validity of the predictive performance of the nomogram in estimating the risk of MVI presence in the training cohort (n=372) (**B**) and validation cohort 1 (n=184) (**C**). External validity of the predictive performance of the nomogram in estimating the risk of MVI presence in the validation cohort 2 (n=31) (**D**).** ROC curve was used to calculate the optimal cutoff values** (**E**). **Heat map for rapid assessment of the risk of MVI presence** (**F**).

All the variables used in this analysis were based on the data obtained preoperatively. The results of the multivariate logistic analysis are presented in a forest plot (**Figure 2E**). Of these, with results reported as the odds ratio (95% *CI*), maximal diameter, ALT, AFP and tumor capsule were independently associated with MVI. These independently associated risk factors were used to form a nomogram that permitted the calculation of the risk of MVI presence (**Figure 3A**). The resulting model was internally validated using the bootstrap validation method. The nomogram demonstrated a good accuracy in estimating the risk of MVI, with an unadjusted C index of 0.739 (95%*CI*, 0.660-0.829) and a bootstrap-corrected C index of 0.745.

In addition, the calibration plots graphically showed a good agreement on the presence of MVI between the risk estimation by the nomogram and the histopathological confirmation of the surgical specimens (**Figure 3B**). In the validation cohort 1 (internal validation cohort), the nomogram displayed a C index of 0.717 (95%*CI*, 0.639-0.795) for the estimation of the MVI risk. There was also a good calibration curve for the risk estimation (**Figure 3C**). In the validation cohort 2 (external validation cohort), the nomogram displayed a C index of 0.709 (95%*CI*, 0.606-0.786) for the estimation of MVI risk (**Figure 3D**).

### Identification the optimal cut-off values of MVI presence

Receiver operating characteristic curve (ROC) analysis was used to determine the optimal cut-off values for the risk of MVI. The area under the ROC curves was 0.739 (*95% CI,* 0.660-0.829) (**Figure 3E**). Nomogram can be interpreted by summing up the points assigned to each variable, and indicated at the top of scale. The total nomogram scores was 168 as the optimal cut-off values. The sensitivity, specificity and consistency rate were used in differentiating the presence from absence of MVI were 65.5%, 82.2% and 80.7% in the training cohort, 66.5%, 88.1% and 83.3% in the validation cohort 1, and 66.7%, 82.1% and 80.6% in the validation cohort 2, respectively (**Supplemental Table 2**). An equivalent heat map for a rapid assessment of the risk of MVI presence was also shown in **Figure 3F**.

## Discussion

Advanced ICC patients are frequently associated with a short-term survival time and high rates of recurrence after the initial treatment, mainly due to the relatively late diagnosis, local invasion, distant metastasis and high recurrence rate. In the current TNM staging system, vascular invasion was incorporated to discriminate the adverse feature of the primary liver tumor by T stage, which commonly indicates a poor prognosis for hepatic cancer patients 13-15.

MVI is a more precise prognostic risk factor in regard of the systemic management and therapy, and its risk grades has been recommended to guide postoperative tumor patients. Cucchetti A et.al^16^ found that preoperative serum alpha-fetoprotein (AFP), tumor number, size, and volume were related to tumor grade and MVI in HCC after hepatic resection and liver transplantation and built a preoperative artificial neural network (ANN) to predict it. Besides, MVI can accurately predict risk of recurrence and survival of patients after HCC resection 17, and has been regarded as an evaluation parameter to the effective of HCC adjuvant transarterial chemoembolization^18^. A predictive and preventable strategy of MVI based on precision medicine will bring profound benefits 19-21.

Lots of previous studies^13^, ^15^, ^22^ reported that tumor diameter, multiple primary tumors, CEA, CA19-9, macro-vascular invasion, lymph node metastasis indicated a relatively poor prognosis of ICC after hepatectomy. MVI, a presentation of potential dissemination in portal system of the liver, is less studied in ICC as a result of the rarity of this cancer. Therefore, a better understanding of the high-risk factors in ICC is necessary to discriminate the aggressive behaviors of ICC but is also essential for guiding management and predicting prognosis.

In the present study, we enrolled a large cohort and demonstrated its prominent prognostic effects on survival and recurrence in ICC patients after curative resection by PSM analysis. We found that the maximal tumor diameter>5 cm, MVI, Age>60 y and CA 19-9≥37 U/ml reflected significant negative prognostic factors of ICC, and they were significantly associated with the OS and DFS by a multivariate Cox regression analysis. In MVI positive patients, the 5-year OS and 5-year DFS are 16.65%, 5.12%, compared with 19.39%, 16.62% in the negative group, respectively. To our knowledge, this is the first study to determine that MVI is an independent risk factor for the prognosis of ICC using an RCT-like method-propensity score matching (PSM). Moreover, we identified a variety of independent risk factors that significantly associated with MVI, namely, as alanine transaminase (ALT), alpha-fetoprotein (AFP), maximal tumor diameter, and tumor capsule, which were then collectively incorporated into the nomogram.

The nomogram was well developed and showed a more accurate value for MVI prediction; consequently, its construction improved the precision of the clinical therapeutic options, such as, preoperative adjuvant chemotherapy and radiotherapy, as well as excision extension. The nomogram was validated by the training cohort C-index 0.739, and as 0.717 and 0.709 for the multi-institutional validation cohorts (Supplemental Table 2), as well as the optimal calibration curves demonstrating agreements between the prediction and actual observation 23-26 (Figure 3, B.C.D).

In summary, these results indicated that tumor diameter>5 cm, incomplete tumor capsule and AFP≥20 ng/ml were associated with an aggressive tumor behavior and increased the possibility of MVI presence in both ICC and HCC 27-33.

For a clinical application of the nomogram, we divided the risk of MVI using 168 as the cutoff value. Patients with a score of 168 or more are a high-risk subgroup of MVI (consistency value, 80.7%). In addition, the existence of MVI is an essential variable to help decide on adjuvant treatments in ICC postoperatively. Therefore, we developed a heat map for a rapid assessment of MVI risk that simplified the process of evaluation with a better visual.

As for the limitations of this study, first is the retrospective design, but we performed a PSM analysis to minimize the systemic and statistical bias to simulate a random controlled trial. Second, the data were derived from two independent institutions in China, and it would be better to validate the results from more centers externally to extend its feasibility. Third, although the nomogram achieved a preferable accuracy, a prospective study is necessary to confirm the reliability of the nomogram. Thus, a prospective multi-center validation may be needed to confirm this prognostic model and the role of MVI in ICC.

## Conclusions

We combined PSM and multivariable Cox regression analyses to determine that MVI is a poor prognostic factor in ICC patients. The finding of a predictive model based on the multicenter data provides an optimal estimation of the MVI risk in patients with ICC, for better predict the clinical prognosis.

## Supplementary Material

Supplementary tables.

## Figures and Tables

**Table 1:** Demographics and clinical characteristics of ICC patients before and after PSM.

Characteristic	Variables	Before PSM, n=556		*P* value	After PSM, n=222 (Match 1 to 4)		*P* value
		Non-MVI	MVI		Non-MVI	MVI	
		n=503(%)	n=53(%)		n=172(%)	n=50(%)	
Gender	Female *vs* Male	212(42.15)/291(57.85)	22(41.51)/31(58.49)	0.929^b^	69(40.12)/103(59.88)	21(42.0)/29(58.0)	0.811^b^
Age(y)	≤60 *vs* >60	286(56.86)/217(43.14)	40(75.47)/13(24.53)	0.009^b^	124(72.09)/48(27.91)	37(74.0)/13(26.0)	0.79^b^
Cirrhotic	No *vs* Yes	377(74.95)/126(25.05)	43(81.13)/10(18.87)	0.319^b^	131(76.16)/41(23.84)	40(80.0)/10(20.0)	0.57^b^
HBsAg	Negative *vs* Positive	312(62.03)/191(37.97)	25(47.17)/28(52.83)	0.035^b^	105(61.05)/67(38.95)	25(50.0)/25(50.0)	0.163^b^
Anti-HCV	Negative *vs* Positive	492(97.81)/11(2.19)	52(98.11)/1(1.89)	0.886^b^	170(98.84)/2(1.16)	49(98.0)/1(2.0)	0.625^b^
AFP(ng/ml)	<20 *vs* ≥20	451(89.66)/52(10.34)	40(75.47)/13(24.53)	0.002^b^	155(90.12)/17(9.88)	39(78.0)/11(22.0)	0.065^b^
CEA(ng/ml)	<5 *vs* ≥5	371(73.76)/132(26.24)	40(75.47)/13(24.53)	0.787^b^	125(72.67)/47(27.33)	37(74.0)/13(26.0)	0.853^b^
CA19-9(U/ml)	<37 *vs* ≥37	212(42.15)/291(57.85)	19(35.85)/34(64.15)	0.376^b^	64(37.21)/108(62.79)	18(36.0)/32(64.0)	0.876^b^
PT(s)	<13 *vs* ≥13	378(75.15)/125(24.85)	43(81.13)/10(18.87)	0.334^b^	129(75.0)/43(25.0)	40(80.0)/10(20.0)	0.465^b^
ALT(U/L)	<35 *vs* ≥35	332(66)/171(34)	24(45.28)/29(54.72)	0.003^b^	81(47.09)/91(52.91)	22(44.0)/28(56.0)	0.699^b^
Ascites	No *vs* Yes	463(92.05)/40(7.95)	45(84.91)/8(15.09)	0.078^b^	158(91.86)/14(8.14)	43(86.0)/7(14.0)	0.213^b^
Tumor number	Solitary *vs* Multiple	430(85.49)/73(14.51)	42(79.25)/11(20.75)	0.227^b^	147(85.47)/25(14.53)	40(80.0)/10(20.0)	0.351^b^
Maximal diameter(cm)	≤5 *vs* >5	221(43.94)/282(56.06)	13(24.53)/40(75.47)	0.006^b^	56(32.56)/116(67.44)	13(26.0)/37(74.0)	0.378^b^
Tumor capsule	No *vs* Yes	429(85.29)/74(14.71)	52(79.25)/1(1.89)	0.009^b^	171(99.42)/1(0.58)	49(98.0)/1(2.0)	0.35^b^
Lymph node invasion	No *vs* Yes	419(83.3)/84(16.7)	42(79.25)/11(20.75)	0.456^b^	144(83.72)/28(16.28)	39(78.0)/11(22.0)	0.349^b^
Tumor differentiation	Ⅰ-Ⅱ *vs* Ⅲ-Ⅳ	275(54.67)/228(45.33)	27(50.94)/26(49.06)	0.604^b^	94(54.65)/78(45.35)	25(50.0)/25(50.0)	0.562^b^
AST(U/L)*	Median(IQR)	26(19,40)	33(25,53)	0.001^a^	29(20,49)	34(25,53)	0.120^a^
Operative year(y)*	Median(IQR)	2007(2005,2010)	2006(2004,2009)	0.056^a^	2007(2005,2010)	2007(2004,2009)	0.346^a^
Propensity Score*	Median(IQR)	0.066(0.043,0.115)	0.124(0.101,0.203)	<0.001^a^	0.115(0.059,0.174)	0.118(0.099,0.203)	0.251^a^

Abbreviations: AST, aspartate aminotransferase; ALT, alanine transaminase; AFP, alpha fetoprotein; CEA, carcinoembryonic antigen; CA19-9, carbohydrate antigen 19-9; PT, prothrombin time; HBsAg, hepatitis B surface antigen; Anti-HCV, anti-hepatitis C virus; MVI, microvascular Invasion; PSM, propensity score matching; IQR, interquartile range. *Skewed distribution: Operative year and Propensity Score are presented as Median (IQR). Caliper: 0.05, Match 1 to 4. a: Mann-Whitney U test(Wilcoxon Rank Sum W Test), b: Pearson Chi-Square Tests, α=0.05.

**Table 2:** Multivariable Cox regression analyses of OS and DFS in ICC patients before and after propensity matched cohort.

Characteristic	Variables	Before PSM				After PSM			
		Multivariate analysis		Multivariate analysis		Multivariate analysis		Multivariate analysis	
		(OS)		(DFS)		(OS)		(DFS)	
		HR (95%*CI*)	*P* value	HR (95%*CI*)	*P* value	HR (95%*CI*)	*P* value	HR (95%*CI*)	*P* value
Gender	Female* vs* Male								
Age(y)	≤60 *vs* >60					0.617(0.398-0.955)	0.03	0.667(0.446-0.998)	0.049
Cirrhotic	No *vs* Yes								
HBsAg	Negative *vs* Positive								
Anti-HCV	Negative *vs* Positive								
AFP(ng/ml)	<20 *vs* ≥20					1.884(1.095-3.242)	0.022		
CEA(ng/ml)	<5 *vs* ≥5	1.300(1.013-1.668)	0.039	1.363(1.072-1.732)	0.011	1.614(1.049-2.483)	0.029		
CA19-9(U/ml)	<37 *vs* ≥37	1.534(1.212-1.941)	<0.001	1.300(1.040-1.626)	0.021	1.665(1.140-2.431)	0.008	1.534(1.069-2.201)	0.02
ALT(U/L)	<35 *vs* ≥35	1.331(1.039-1.705)	0.023						
PT(s)	<13 *vs* ≥13								
AST(U/L)*	Median(IQR)								
Ascites	No *vs* Yes					1.946(1.095-3.459)	0.023		
Tumor number	Solitary *vs* Multiple								
Maximal diameter(cm)	≤5 *vs* >5	1.351(1.075-1.698)	0.010	1.440(1.158-1.790)	0.001	2.103(1.219-3.629)	0.008	1.485(1.009-2.183)	0.045
MVI	No *vs* Yes			1.414(1.012-1.975)	0.042	1.635(1.405-1.993)	0.04	1.596(1.077-2.366)	0.02
Tumor differentiation	Ⅰ-Ⅱ *vs* Ⅲ-Ⅳ	1.388(1.120-1.720)	0.003	1.338(1.090-1.644)	0.005			1.401(1.013-1.938)	0.042
Lymph node invasion	No *vs* Yes	2.068(1.565-2.732)	<0.001	1.477(1.123-1.943)	0.005				
Tumor capsule	No *vs* Yes								
Operative year*	Median(IQR)								

Abbreviations: AST, aspartate aminotransferase; ALT, alanine. transaminase; AFP, alpha fetoprotein; CEA, carcinoembryonic antigen; CA19-9, carbohydrate antigen 19-9; PT, prothrombin time; HBsAg, hepatitis B surface antigen; Anti-HCV, anti-hepatitis C virus; MVI, microvascular Invasion; PSM, propensity score matching; OS, overall survival; DFS, disease-free survival; HR, hazard ratio; IQR, interquartile range. Method=Enter, α=0.05.

## Abbreviations

AST: Aspartate aminotransferase
ALT: Alanine transaminase
AFP: Alpha-fetoprotein
Anti-HCV: Anti-hepatitis C virus
CEA: Carcinoembryonic antigen
CA19-9: Carbohydrate antigen 19-9
PT: Prothrombin time
HBsAg: Hepatitis B surface antigen
MVI: Microvascular Invasion
PSM: Propensity score matching
PS: Propensity score
IQR: Interquartile range
OS: Overall survival
DFS: Disease-free survival
HR: Hazard ratio
ICC: Intrahepatic cholangiocarcinoma
CCAs: Cholangiocarcinomas
ECC: Extrahepatic cholangiocarcinoma
HCC: Hepatocellular carcinoma
RFA: Radiofrequency ablation
TACE: Transarterial chemoembolization
ROC: Receiver operating characteristic curve
OR: odds ratio
