# Supplementary material for: Prognostic Value and Predication Model of Microvascular Invasion in Patients with Intrahepatic Cholangiocarcinoma
Source: J Cancer. 2019 Sep 7;10(22):5575–84. doi: 10.7150/jca.32199 (PMC6775679; doi:10.7150/jca.32199)
Supplement: Supplementary file 1 — Supplementary tables. [file jcav10p5575s1.pdf]

Supplemental Table 1 Univariable Cox regression analyses of OS and DFS in ICC patients before and after propensity matched cohort.

Characteristic	Variables	Before PSM				After PSM			
		Univariate analysis (OS)		Univariate analysis (DFS)		Univariate analysis (OS)		Univariate analysis (DFS)	
		HR(95%*CI*)	*P* value	HR(95%*CI*)	*P* value	HR(95%*CI*)	*P* value	HR(95%*CI*)	*P* value
MVI	No *vs* Yes	1.177(0.834-1.663)	0.354	1.670(1.223-2.281)	0.001	0.961(0.652-1.417)	0.842	1.715(1.204-2.442)	0.003

Abbreviations: MVI, microvascular Invasion; OS, overall survival; DFS, disease-free survival; HR, hazard ratio; PSM, propensity score matching.

Method=Enter, α*=0.05.*

Supplemental Table 2 Accuracy of the Prediction Score of the Nomogram for Estimating the Risk of MVI Presence.

Variable	Value (95% *CI*)		
	Training Cohort	Validation Cohort 1	Validation Cohort 2
Area under ROC curve, concordance index	0.739 (0.660-0.829)	0.717 (0.639-0.795)	0.709(0.606-0.786)
Cutoff score	168	168	168
Period prevalence rate, %	8.9	11.6	9.7
Sensitivity, Se, %	65.5(45.7-82.1)	66.5(51.2-82.3)	66.7(59.4-99.2)
Specificity, Sp, %	82.2(77.3-86.3)	88.1(84.2-91.4)	82.1(63.1-93.9)
Positive predictive value, PPV, %	26.4(16.7-38.1)	33.9(22.1-47.4)	28.6(23.7-71.0)
Negative predictive value, NPV, %	96.1(92.9-98.1)	92.7(89.2-95.3)	95.8(78.9-99.9)
Consistency rate, %	80.7(76.0-84.8)	83.3(79.1-87.0)	80.6(62.5-92.5)

Abbreviations: MVI, microvascular invasion; ROC, receiver operating characteristic. Patients who had a nomogram score of less than 168 or 168 or greater were considered to have low or high risks of MVI presence, respectively.
